# Role of the Adjacent Stroma Cells in Prostate Cancer Development and Progression: Synergy between TGF-*β* and IGF Signaling

**DOI:** 10.1155/2014/502093

**Published:** 2014-06-25

**Authors:** Chung Lee, Zhenyu Jia, Farah Rahmatpanah, Qiang Zhang, Xiaolin Zi, Michael McClelland, Dan Mercola

**Affiliations:** ^1^Department of Urology, Northwestern University Feinberg School of Medicine, Tarry Building, Room 16-733, 303 East Chicago Avenue, Chicago, IL 60611, USA; ^2^Department of Urology, University of California at Irvine, Medical Center, Orange, CA 92868, USA; ^3^Department of Pathology and Laboratory Medicine, University of California at Irvine, Medical Surgery Building I, Room 168, Irvine, CA 92697, USA; ^4^Department of Surgery, NorthShore University HealthSystem, Evanston, IL 60201, USA; ^5^Department of Statistics, University of Akron, Akron, OH 44325, USA; ^6^Department of Family and Community Medicine, Northeast Ohio Medical University, Rootstown, OH 44272, USA

## Abstract

This review postulates the role of transforming growth factor-beta (TGF-*β*) and insulin-like growth factor (IGF-I/IGF-II) signaling in stromal cells during prostate carcinogenesis and progression. It is known that stromal cells have a reciprocal relationship to the adjacent epithelial cells in the maintenance of structural and functional integrity of the prostate. An interaction between TGF-*β* and IGF signaling occupies a central part in this stromal-epithelial interaction. An increase in TGF-*β* and IGF signaling will set off the imbalance of this relationship and will lead to cancer development. A continuous input from TGF-*β* and IGF in the tumor microenvironment will result in cancer progression. Understanding of these events can help prevention, diagnosis, and therapy of prostate cancer.

## 1. Introduction

Carcinogenesis is a multistep process which begins with initiation followed by promotion and progression [1]. The adjacent stromal cells play an important role in this process [2–4]. The signaling events of transforming growth factor-*β* (TGF-*β*) and insulin-like growth factor (IGF-I and IGF-II) in stromal cells occupy a central part in the stromal-epithelial interaction during cancer development and progression [5–9]. In this review, we will propose a hypothesis describing a synergistic role between TGF-*β* and IGF signaling in stromal-epithelial interaction in prostate cancer.

## 2. Biology of TGF-***β*** Signaling

TGF-*β* represents a family of pleiotropic growth factors with diverse functions, such as embryonic development, wound healing, organ development, immunomodulation, and cancer progression [10–12]. There are three known mammalian isoforms of TGF-*β* (TGF-*β*1, -*β*2, and -*β*3) with significant homology and similarities in function. The biological effect of TGF-*β* is mediated through type I and type II receptors [13, 14]. The downstream events include both Smad and non-Smad signaling pathways [15–17]. The relative importance and interplay of these pathways of TGF-*β* signaling are still under investigation [18–21]. In general, events mediated through the Smad pathways are mainly related to growth arrest and apoptosis, while those mediated through the non-Smad pathways are mainly related to cell proliferation and migration [17].

## 3. Biology of IGF Signaling

The IGF axis consists of two ligands (IGF-I and IGF-II), two cell surface receptors (IGF-IR and IGF-IIR), 6 binding proteins (IGFBP-1 to 6), and a group of IGFBP degrading enzymes [22–24]. Among IGFBPs, IGFBP-3 is the most abundant in the prostate and is able to bind IGF-I and thus controls the amount of available IGF-I to interact with IGF-IR in target cells. IGF-IR is a tyrosine kinase receptor. Upon engagement with IGF-I or IGF-II, IGF-IR is activated by phosphorylation and activates downstream mitogenic signals, including MAPK and PI3K. IGF-IIR does not have the intracellular kinase domain and its role in cellular proliferation remains unclear [25]. An important aspect of IGF signaling in prostate cancer development and progression is that it is able to activate androgen receptor nuclear translocation in the absence of androgen [26–28]. Epidemiologic and laboratory evidence strongly suggests that elevated IGF-I levels are associated with increased risk of prostate cancer [5, 25, 29].

## 4. Stromal-Epithelial Interaction in the Normal Prostate: The Prostatic Ductal System

During embryogenesis, epithelial cells in the ectoderm change into mesenchymal cells, which migrate through the primitive streak and insert themselves between the ectoderm and the endoderm. Some of these mesenchymal cells will engage in the establishment and maintenance of a lifelong relationship with the epithelial cells. The reciprocal relationship between the stroma and epithelia in the urogenital sinus has created a unique microenvironment which directed the urogenital sinus to be destined to the development of the prostate [30].

In the normal prostate, TGF-*β* is a gate keeper to maintain cellular homeostasis and structural integrity. The cross talk between two cellular components is mainly centered on the TGF-*β* and IGF signaling. The adult prostate is arranged as individual ductal systems, consisting of the proximal, intermediate, and distal regions [31, 32]. The integrity of these regions is maintained in a homeostasis state through a tightly regulated TGF-*β* signaling cross talk between the stromal and epithelial compartments [33–35]. The epithelial compartment in the distal region contains proliferative cells; the epithelial cells in the proximal region are undergoing apoptosis. The majority of the epithelial cells in the ductal system are located in the intermediate region and are in a differentiated, proliferative quiescent state [31, 32]. The adjacent stromal compartment consists of fibroblasts and smooth muscle cells. Smooth muscle cells are concentrated in the proximal region as they produce high levels of TGF-*β*, while the fibroblasts are lined in the distal region and produce little or no TGF-*β* [32, 34]. This regional differential production of TGF-*β* is critical, in that TGF-*β* is a potent inhibitor for proliferation in the adjacent epithelial cells. The maintenance of cellular homeostasis within this ductal system implemented by a regional variation in stromal-epithelial cross talk is mediated by a corresponding regional TGF-*β* signaling. A disturbance of this delicate balance between the stroma and the epithelia will result in abnormal growth of the prostate, such as benign prostatic hyperplasia and prostate cancer, when the IGF signaling system derived from the stromal cells through a paracrine fashion to play a synergistic role [23, 29].

## 5. A Biphasic Effect of TGF-***β*** on Normal Prostate Stromal and Epithelial Cells

TGF-*β*, under normal physiological conditions, is a gate keeper to maintain cellular homeostasis, including the maintenance of the normal integrity of the prostate. A common notion is that TGF-*β* is inhibitory to cell growth and proliferation in normal cells. This notion needs to be modified. Results of our studies have demonstrated a biphasic effect of TGF-*β* on both prostatic stromal [36] and epithelial cells [37]. At a low dose (0.1 *η*g/mL), TGF-*β* can stimulate cell proliferation through the induction of mitogenic factors [37, 38], while, at a high dose (10 *η*g/mL), TGF-*β* inhibits cell proliferation through the induction of CDK inhibitors and inactivation of Erk [36, 37]. The biphasic effect of TGF-*β* offers a mechanism to maintain cellular homeostasis under normal physiological conditions.

## 6. The TGF-***β*** Paradox and Differential Effect of TGF-***β*** between Benign and Cancer Cells

It has been known that the effect of TGF-*β* is different between benign and cancer cells. TGF-*β* mediates growth inhibition and apoptosis in benign cells but facilitates progression and metastasis in cancer cells [37]. The mechanism for this differential effect of TGF-*β* remains unclear. Results of our studies have observed a differential effect of TGF-*β* on Erk activation between benign and cancer cells which have provided a partial answer to this paradox. At low dose of TGF-*β* (0.1 *η*g/mL), both benign cells and cancer cells undergo Erk activation and induction of TGF-*β* production [37]. At high doses (10 *η*g/mL), TGF-*β* inhibits Erk activation in benign cells, but, in cancer cells, TGF-*β* continuously mediates Erk activation and induction of TGF-*β* production [37, 39]. This differential effect of TGF-*β* constitutes a critical event in the TGF-*β* paradox and creates a unique tumor microenvironment that sets off a vicious cycle to promote tumor progression. In the following paragraphs, we will discuss the role of adjacent stromal cells in prostate cancer development, progression, and metastasis.

## 7. Reactive Stroma—Myofibroblasts or Cancer-Associated Fibroblasts (CAFs)

Fibroblast-to-myofibroblast transdifferentiation is a hallmark of benign prostatic hyperplasia and prostate cancer [40–43]. The presence of myofibroblasts is known to promote proliferation of the adjacent epithelial cells in the prostate [42, 43]. Unlike the normal fibroblasts which do not produce TGF-*β*, CAFs produce large amounts of TGF-*β*, which is the inducing stimulus for growth of the adjacent epithelial cells [43, 44]. Direct stimulation of fibroblasts with TGF-*β* can induce the transdifferentiation of fibroblasts to myofibroblasts with Erk activation [41], which may also require activation of Smad3 [40]. Myofibroblasts express and secrete proinvasive factors significantly increasing the invasive capacity of tumor cells via paracrine mechanisms. Reactive stroma can also be induced by down regulating TGF-*β* receptors, which will lead to an increased production of TGF-*β*. This is illustrated by the introduction of a dominant negative type II TGF-*β* receptor into prostate stromal cells resulting in an increase in the simultaneous expression of vimentin and *α*-smooth muscle actin (definition of myofibroblasts) through the activation of the AKT pathway [44]. Conversely, the myofibroblast phenotype can be detected adjacent to established cancer [45]. In the present review, we will use the term cancer-associated fibroblasts (CAFs) in place of reactive stroma or myofibroblasts.

## 8. Role of the Adjacent Stroma in Prostate Carcinogenesis

Under normal physiological conditions, the homeostasis of the normal prostate is carefully maintained by the well-orchestrated stromal-epithelial cross talk through a tightly regulated TGF-*β* signaling in a paracrine fashion [32]. An imbalance in TGF-*β* signaling within this normal stromal-epithelial interaction will result in abnormal growth of the prostate. During prostatic carcinogenesis in rats and humans, the adjacent stroma undergoes progressive loss in smooth muscle with the appearance of CAFs [43, 46, 47]. Perhaps the best example is the report by Bhowmick et al. in 2004 [48] in which they introduced a dominant negative type II TGF-*β* receptor into prostate stromal cells resulting in the loss of TGF-*β* responsiveness in these stromal cells, which assumed a myofibroblast phenotype. These genetically altered fibroblasts also produce elevated TGF-*β* [49]. In retrospect, it becomes apparent that these CAF cells not only produced an increased level of TGF-*β*, but also produced an increased level of IGF-I [50, 51]. We hypothesize that under the combined influence of elevated TGF-*β* and IGF-I, along with the input from androgen/estrogen signaling, the adjacent epithelial cells eventually developed into prostate cancer. This assumption implies that an increased TGF-*β* and IGF signaling in the adjacent stromal cells can lead to the development of prostate cancer [44]. Indeed, it has been shown that myofibroblasts contained a reduced level of TGF-*β* receptors when compared to that in the normal fibroblasts [52]. Further, it is interesting that the CAF cells with aberrant TGF-*β* signaling events can also interact with the neighboring normal stromal cells to jointly impact prostate carcinogenesis [44]. In conclusion, an imbalance in TGF-*β* signaling by a reduction in TGF-*β* sensitivity and an increased production of TGF-*β* coupled with an increased IGF-I signaling in the CAF cells is able to bring about malignant transformation in the adjacent epithelial cells in the prostate [29, 48, 49].

We postulate that an intricate interaction between TGF-*β* and IGF-I signaling in prostate CAF cells has created a unique microenvironment which is conducive for cancer development and progression in the adjacent epithelial cells. Prostate CAF cells are able to produce IGF-I in response to TGF-*β* [50, 51]. Likewise, CAF cells are able to produce TGF-*β*1 in response to IGF-I [53]. Such an interaction has created a positive feedback loop to stimulate the adjacent epithelial cells to undergo proliferation and carcinogenesis [29].

## 9. Role of the Adjacent Stroma in Tumor Progression 

**Figure 1:**
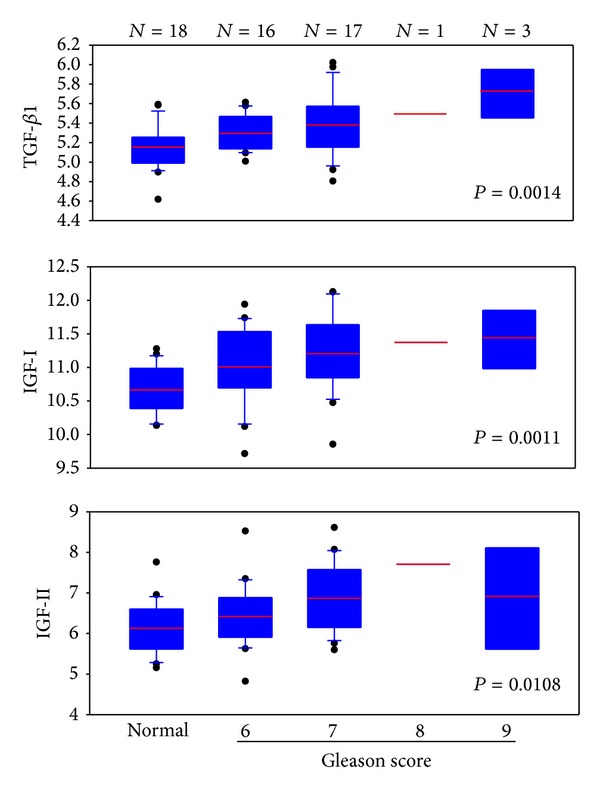
In a study of diagnosis of prostate cancer using stromal signatures [61], we identified 18 normal subjects and 37 prostate cancer patients in whom the biopsy contained no cancer elements. Microarray analysis was performed on an Affymetrix U133A 2.0 array platform. We extracted the data for TGF-*β*1, IGF-I, and IGF-II for these 55 cases. The expression intensities were normalized against those for GAPDH. The resulting relative values for each growth factor were plotted on the *y*-axis. Results indicated that, in all three growth factors, the values for the normal subjects were relatively low. The values for cancer cases increased and were correlated with Gleason score, suggesting that the expression of these growth factors is associated with cancer progression.

In prostate cancer, both the cancer cells and the adjacent CAFs will mediate oncogenic signals to fuel the cancer progression and metastasis [45, 46, 54]. Again, an aberrant TGF-*β* signaling in both the CAF and the adjacent epithelial compartment sets off a vicious cycle for progression and metastasis [55–57]. Many paracrine signals promote prostate cancer cell adhesion in the bone matrix. Fibroblast-to-myofibroblast transdifferentiation can lead to many activities of TGF-*β* mediated events in cancer. These events include changes in cytokine balances, EMC proteins, proteases, and IGF-I production, resulting in cancer invasion and ectopic survival, angiogenesis, and evasion of host immune surveillance program [55, 57–59]. In addition to contributing to cytokines, modified ECM, proteases, and protease inhibitors, myofibroblasts themselves are able to invade into cancer cell compartment [60]. In a recent paper [61], we observed an increased expression of TGF-*β*1, IGF-I, and IGF-II in the stromal cells adjacent to prostate cancer. These levels correlated with the Gleason score of the disease, suggesting that expression of TGF-*β*1, IGF-I, and IGF-II is associated with cancer progression (Figure 1). In fact, genetic signatures in the stroma can be used to detect the presence of malignancy in adjacent epithelial cells [61].

## 10. Androgen Receptor (AR) and Androgen Action

AR in the stroma is known to play a role in normal and malignant prostate [30, 46]. AR is detected in both epithelial and stromal cells, in cancer, as well as in benign prostate [62]. Both TGF-*β* and IGF-I can interact with AR function in the prostate. IGF signaling can result in AR translocation to the nucleus in the absence of androgen [26–28]. Androgen can also upregulate IGF-I production from prostate stromal cells [63]. Results of many studies indicated that AR signaling can negatively regulate TGF-*β* signaling through the negative AR response element [64, 65], while TGF-*β* signaling represses AR signaling through Smad3 [66]. However, corroboration between TGF-*β* and AR signaling in prostate cancer has also been reported [67, 68]. This discrepancy may be attributed to a differential interaction between activation of AR coregulator through Samd3 [66, 69] and DNA acetylation through Samd4 [68, 69].

## 11. Estrogen Receptors (ER) and Estrogen Action

17*β*-Estradiol is a natural estrogen produced in both males and females. It mediates its action through interaction with estrogen receptor (ER). There are three types of estrogen receptors, ER*α*, ER*β*, and GPR30 [70]. ER*α* and GPR30 promote proliferation, whereas ER*β* has proapoptotic and prodifferentiating functions [70, 71]. Prostate stromal cells contain mainly ER*α* and GPR30 [69]. E2 stimulates production of TGF-*β* in prostate stromal cells through ER*α* [72]. E2 can also upregulate IGF-IR through a nongenotropic pathway in prostate cancer cells [73]. A recent report indicated that expression of ER*α* in CAF would suppress prostate cancer invasion [74]. Since cancer cell invasion is mediated by TGF-*β* signaling, this observation supports the notion that there is a negative interaction between ER-*α* and TGF-*β* signaling [75]. A unique property of estrogen is its ability to be metabolized to 2-catechol estrogen, which may react with DNA to form depurinated adducts [76, 77]. These DNA adducts will lead to cancer initiation. This is also consistent with the report that high circulating estrogen is associated with increased incidence of prostate cancer [78].

## 12. Combination of Androgen and Estrogen in Prostate Carcinogenesis

The best example of prostate cancer generation is treating Noble rats with a combination of androgen and estrogen and tumors were developed in the dorsal lobe of the prostate [29, 79, 80]. Prostate cancer can also be generated by a combination of stromal cells with basal prostatic epithelial cells with both estrogen and testosterone [81], while testosterone alone would not induce prostate cancer. A classical study by [82] has illustrated the significance of a combination of androgen and estrogen affecting the stromal cells to promote prostate cancer development. In untreated mouse hosts, UGM + BPH-1 recombinants produced solid branched epithelial cords and ductal structures exhibiting benign growth. In T + E2-treated hosts, UGM + BPH-1 recombinants formed invasive carcinomas. BPH-1 cells lack androgen and estrogen receptors, whereas rat UGM expresses both of these receptors. IGF-I signaling is responsible for carcinogenesis as elicited by T + E2 in the stromal microenvironment [29]. DNA adducts are observed in Noble rats [83]. Based on this review, we postulate that the effect of androgen/estrogen causes DNA damage and is the initiation step for prostate carcinogenesis, while the impact of TGF-*β* and IGF signaling is the promotion step leading to cancer development and progression.

## 13. Conclusions: Targeting the Stromal Cells for Cancer Diagnosis, Prevention, and Therapy

**Figure 2:**
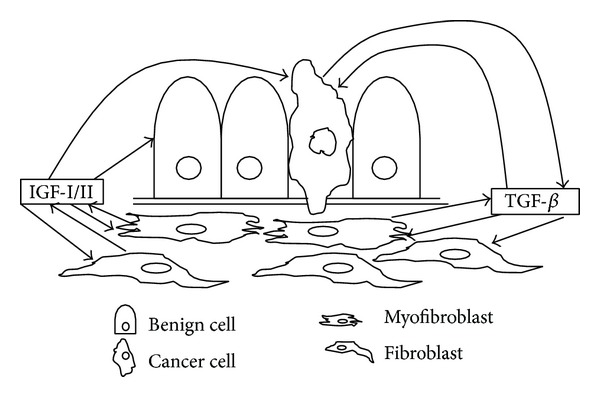
A simplified scheme depicting our hypothesis of the role of stromal-epithelial interaction in prostate cancer development and progression. Under the normal condition, signaling events between TGF-*β* and IGF are tightly regulated keeping the epithelial cells under a homeostatic balance. A reduction in TBRs in the stromal cells will result in an increase in IGF production, which has a proliferative effect on the prostate epithelial cells which have undergone a cancer initiation process as a result of T + E2. TGF-*β* and IGF in the stromal cells adjacent to prostate epithelial cells will perpetuate a vicious cycle to promote cancer progression.

Based on the above discussion, we understand that contributions from the adjacent stromal cells can control tumor development and progression. As depicted in Figure 2, we propose the hypothesis that an imbalance in signaling between TGF-*β* and IGF in the stromal cells adjacent to prostate epithelial cells is responsible for the carcinogenesis process which perpetuates this imbalance in a vicious cycle to further promote cancer progression. Initially, the carcinogenesis initiation step is thought to be the result of androgen/estrogen action. Subsequently, the carcinogenesis promotion step is thought to be triggered by the TGF-*β*/IGF signaling. A downregulation of TGF-*β* receptors in the stromal cells will result in an increase in TGF-*β* expression. An increased level of TGF-*β* in the microenvironment will induce the expression of IGF-I/IFG-II and IGFBP-3. IGFBP-3 will regulate the bioavailability of IGF-I/IGF-II. However, IGFBPs can be degraded by PSA and MMPs [23, 50, 84], as a result of TGF-*β* and androgen receptor signaling, leaving the activation of the mitogenic and carcinogenic action of IFG signaling to the adjacent epithelial cells. Once the cancer is developed, these cells will produce an increased level of TGF-*β*, which will fuel the adjacent stromal cells to manifest additional TGF-*β*/IGF synergy leading to further cancer progression. With this hypothesis, it will be possible for us to consider targeting a combination of TGF-*β* and IGF signaling for treatment of prostate cancer. Inhibitors that can block the signals of TGF-*β* and IGF are either available or currently in clinical trials [85–87].
